# Editorial: Women in sports and exercise: public health and promotion

**DOI:** 10.3389/fpubh.2026.1936420

**Published:** 2026-08-12

**Authors:** Bojan Masanovic, Szabolcs Halasi, Dusan Stupar, Gonul Babayigit Irez, Catalina Casaru

**Affiliations:** 1 Faculty for Sport and Physical Education, University of Montenegro, Niksic, Montenegro; 2 Western Balkan Sport Innovation Lab, Podgorica, Montenegro; 3 Hungarian Language Teacher Training Faculty, University of Novi Sad, Subotica, Serbia; 4 Faculty of Sport and Psychology, TIMS, Educons University, Novi Sad, Serbia; 5 Department of Kinesiology, University of West Alabama, Livingston, AL, United States; 6 Faculty of Sport Sciences, Mugla Sitki Kocman University, Mugla, Türkiye

**Keywords:** exercise interventions, gender equity, health promotions, monitoring systems, public health, sports performance, women in sports

## 1 Introduction

Although participation in sport and physical exercise has increased substantially over recent decades (1), research in this field has remained predominantly focused on men (2). As a result, the evidence base regarding women's specific physiological mechanisms, training responses, recovery patterns, and performance-related factors remains limited (3). Strengthening the scientific evidence on women's participation in sport and physical activity should therefore be regarded as a public health and research priority, as investigating sex differences in motivation, participation, and engagement in physical activity can provide valuable insights for the development of more inclusive and effective health and sport policies. Consequently, there is a growing need for research examining women's physiological responses to exercise (4) across different age groups and settings, while also identifying strategies to improve physical activity levels, physical fitness, and overall health outcomes. This Research Topic was developed to advance and consolidate the scientific evidence on women in sport and exercise, providing a stronger foundation for the development of more effective strategies to promote physical activity and public health within this population.

## 2 Contribution to the field

The aim of this Research Topic was to compile emerging evidence on women in sport and exercise across different stages of life and to provide both theoretical and practical insights that may contribute to the development of more effective strategies for promoting physical activity and public health among women. This Research Topic comprises seventeen articles, including fourteen Original Research papers (ten cross-sectional studies, two randomized controlled trials, one prospective injury surveillance study, and one secondary data analysis of the GBD 2021 database) and three Review papers (two meta-analyses and one narrative review). Collectively, these studies provide a comprehensive overview of current evidence and substantially advance knowledge in this rapidly evolving field. Overall, the studies included approximately 496,493 participants (including 1,220 men) from more than 204 countries and territories, with 30 countries explicitly represented: China, New Zealand, England, France, Canada, Australia, Wales, Ireland, Scotland, Italy, the United States, Japan, South Africa, Fiji, Samoa, Spain, the Netherlands, Kazakhstan, Colombia, Madagascar, Sweden, South Korea, Brazil, Türkiye, Jordan, Iran, Nigeria, Pakistan, Malaysia, and India. Participants ranged in age from 14 years to over 80 years.

The studies included in this Research Topic covered a broad range of methodological approaches, research themes, and age groups, allowing for several possible classification frameworks. To provide a clear and coherent synthesis of the findings, we organized the studies into four thematic categories: (1) psychosocial determinants of women's participation in sport and physical activity, (2) physical activity and physical and mental health outcomes, (3) exercise interventions and physical performance, and (4) injury and fracture epidemiology.

The first group comprised six studies addressing the psychosocial and behavioral determinants (five cross-sectional studies and one Narrative Review). Their findings highlight: stronger internal motivation is associated with reduced job burnout among female physical education teachers (Liu et al.); viewing exercise as an important part of life and deriving pleasure and a sense of accomplishment from it contribute to sport participation (Chen et al.); women experiencing stress related to their gender roles exhibit slightly higher motivation for sport participation (Yalcin et al.); smartphone addiction negatively impacts active lifestyle habits and increases the tendency toward sedentary behavior among young women (Altinisik et al.); female college students' participation in sport is influenced by gender norms, cultural expectations, and social and environmental factors (Zhou and Liu); and addressing social anxiety should be considered an important component of interventions designed to promote physical activity among women (Huang et al.).

The second group consisted of four cross-sectional studies examining the associations between physical activity and physical and mental health. Based on their results, it can be concluded: physical activity and enjoyment of physical activity were positively associated with subjective and mental wellbeing among infertile women and female students, respectively (Liu et al.; Karakullukcu et al.); women who participated in sports showed significantly lower endorsement of gender inequality ideology compared to non-participants (Fang et al.); and regular yoga practice was associated with improved psychological wellbeing, stress management, self- and body awareness, pain reduction, posture, flexibility, quality of life, and reduced reliance on medication among women with spinal disorders (Akyol et al.).

The third group included five studies examining exercise interventions and physical performance (two meta-analyses, two randomized controlled trials and one cross-sectional study). Their results demonstrated: resistance training effectively enhanced muscle strength and physical function in older women with sarcopenia (Zhou et al.); maximum sprint speed and Wingate mean power effectively differentiated starting from substitute female rugby players (Gençoglu et al.); high-intensity interval resistance training improved body composition and lipid metabolism by reducing body weight, body fat, triglycerides, total cholesterol, and LDL levels while increasing HDL cholesterol (Yamaner et al.); while ballistic exercise produced rapid initial improvements in countermovement jump and sprint performance that plateaued over time, complex training resulted in slower but continuous improvements in jumping, sprinting, and maximal strength, leading to superior long-term strength development (Wang S. et al.); and plaza dancing improved body composition and cardiopulmonary health by reducing body weight, BMI, body fat, resting heart rate, blood pressure, cholesterol, and triglycerides while increasing vital capacity (Yao et al.).

The fourth group consisted of two studies (one prospective injury surveillance study, and one secondary data analysis of the GBD 2021 database) examining the Injury epidemiology. The available evidence indicates: women's international rugby had an overall injury incidence of 49.5 injuries per 1,000 player-match-hours, with concussion and knee-ligament injuries being the most common, and two-thirds of injuries occurring in the second half of matches, suggesting fatigue as a major risk factor (Fuller and Taylor); the burden of fall-induced hip fractures among perimenopausal women increased between 1990 and 2021, particularly among women aged 50–54 years and in countries with a high sociodemographic index, although the risk of hip fractures generally declined over time (Wang Z. et al.).

## 3 Conclusion

This Research Topic, comprising seventeen articles, strengthens the growing evidence on the benefits of sport and physical activity for women's health, physical performance, and wellbeing, while addressing important knowledge gaps in women-focused sport and exercise research. Such evidence is essential for developing evidence-based recommendations and interventions tailored to women's specific needs (5, 6). Collectively, the studies included in this Research Topic demonstrate that effective promotion of women's participation in sport and physical activity requires more than simply encouraging exercise. It also requires understanding the psychosocial factors that influence participation, recognizing the broad physical and mental health benefits of physical activity, implementing evidence-based exercise interventions that optimize health and performance, and considering injury patterns and age-specific health risks when designing prevention strategies.

Ultimately, we hope that the evidence and perspectives presented in this Research Topic will stimulate new research, inspire innovative interventions, and contribute to the development of more effective strategies for promoting women's participation in sport and physical activity, thereby improving women's health, performance, and wellbeing across the lifespan.
